# A Unique Trinucleotide-Bloc Mutation-Based Two SARS-CoV-2 Genotypes with Potential Pathogenic Impacts

**DOI:** 10.1155/2022/5618222

**Published:** 2022-07-19

**Authors:** Mustak Ibn Ayub

**Affiliations:** ^1^Department of Genetic Engineering and Biotechnology, University of Dhaka, Dhaka, Bangladesh; ^2^Cancer Care and Research Trust Bangladesh (CCRTB), Dhaka, Bangladesh

## Abstract

SARS-CoV-2, the novel coronavirus behind the COVID-19 pandemic, is acquiring new mutations in its genome. Although some mutations provide benefits to the virus against human immune response, others may result in their reduced pathogenicity and virulence. By analyzing more than 3000 high-coverage, complete sequences deposited in the GISAID database up to April 2020, here I report the uniqueness of the 28881–28883: GGG > AAC trinucleotide-bloc mutation in the SARS-CoV-2 genome that results in two substrains, described here as SARS-CoV-2g (28881–28883: GGG genotype) and SARS-CoV-2a (28881–28883: AAC genotype). Computational analysis and literature review suggest that this bloc mutation would bring 203–204: RG (arginine-glycine)>KR (lysine-arginine) amino acid changes in the nucleocapsid (N) protein affecting the SR (serine-arginine)-rich motif of the protein, a critical region for the transcription of viral RNA and replication of the virus. Thus, 28881–28883: GGG > AAC bloc mutation is expected to modulate the pathogenicity of SARS-CoV-2. These analyses suggest that SARS-CoV-2 has evolved into SARS-CoV-2a affecting COVID-19 infectivity and severity. To confirm these assumptions, retrospective and prospective epidemiological studies should be conducted in different countries to understand the course of pathogenicity of SARS-CoV-2a and SARS-CoV-2g. Laboratory research should focus on the bloc mutation to understand its true impacts on the course of the pandemic. Potential drug and vaccine development should also keep the 28881–28883 region of the N protein under consideration.

## 1. Introduction

SARS-CoV-2 is a positive-stranded RNA virus and has already infected millions of people around the globe. With a genome size of ∼30000 bases and very high infectivity, the virus has already amassed numerous changes in its genome and is acquiring more.

The genome organization of SARS-CoV-2 is similar to other coronaviruses [1]. It has Open Reading Frames (ORFs) common to all beta-coronaviruses which includes ORF1ab responsible for most of the enzymatic proteins, surface glycoproteins (S), envelope proteins (E), membrane proteins (*M*), and nucleocapsid proteins (N). There are also nonstructural proteins expressed mostly from ORF3a, ORF6a, ORFF7a, and ORF8a. The reference genome of SARS-CoV-2 includes ORF10a as part of its genome as shown in Table 1.

Notably, whole-genome sequencing of SARS-CoV-2 and deposition to the public database have been progressing at an unprecedented pace since the beginning of this outbreak.

This is one of the initial studies conducted on the SARS-CoV-2 genomes (doi:10.20944/preprints202004.0337.v1) where ∼3000 whole-genome sequences available on the GISAID database up to April 2020 were analyzed. This study has proposed that a unique trinucleotide-bloc mutation, 28881–28883: GGG > AAC has given rise to a new subtype of SARS-CoV-2 with potential impacts on the course of the COVID-19 pandemic. This bloc mutation is mapped within the nucleocapsid (N) gene according to the SARS-CoV-2 reference genome. The nucleocapsid (N) protein has three dynamic disordered regions that contain putative transiently helical binding motifs. The full-length N protein is a flexible and multivalent RNA-binding protein which is crucial for viral replication and genome packaging [2]. N protein plays a critical role to assemble the coronavirus RNA genome and creates a shell around the enclosed nucleic acid [3]. It also interacts with the viral membrane protein during viral assembly, assists in RNA synthesis, folding, and virus budding. The protein also affects host cell responses to viral infection, including cell cycle regulation and immune responses modulation [4].

The 28881–28883: GGG > AAC mutation affects the SR (serine-arginine)-rich domain of the N protein. Previously in SARS-CoV-1, the closest neighbor to SARS-CoV-2, it has been shown that experimentally introduced deletion in the SSRSSSRSRGNSR region of the SR-rich motif significantly reduces the infectious virions [5]. The 28881–28883: GGG > AAC mutation affects the location adjacent to the aforementioned region and so is expected to impact the pathogenicity of SARS-CoV-2 in a similar manner.

History of previous infections suggests that viruses with different pathogenicities were acquired through mutations [6, 7]. Although hundreds of mutations have been reported in the SARS-CoV-2 genome to date, the trinucleotide-bloc mutation reported and characterized in this study has unique features with potential impact on the pathogenicity of the virus.

This study suggests that by monitoring the prevalence of the SARS-CoV-2a and SARS-CoV-2g strains, countries may track the course of the COVID-19 pandemic. Potential drugs can be designed to target the SR-rich motif of the N protein to curb the pathogenicity of SARS-CoV-2.

## 2. Materials and Methods

### 2.1. Data Collection

This study had been conducted based on an analysis of the ∼3000 whole-genome sequences of SARS-CoV-2 from the GISAID database up to April 2020. Not only the country-wise information was considered, but the study also took the advantage of region-based sequences deposited in the database. COVID-19 trackers, such as Microsoft Bing and Statista website, were frequently used to get information about COVID-19 cases in the regions from where virus genome sequences have been deposited.

Firstly, the study looked at the early sequences deposited from Italy, one of the worst affected countries where the death toll was very high. In Italy, Lombardy has experienced most cases and deaths from COVID-19, a big contrast with Abruzzo, which has a very low number of COVID-19 cases and deaths. When the region specific-sequences deposited in the GISAID database were examined, it was found that SARS-CoV-2 from Abruzzo stands out compared to other regions of Italy, especially of Lombardy. The most striking difference was the change in a bloc of three nucleotides at 28881–28883 location where a GGG > AAC change has occurred. Sequences from Abruzzo were predominantly 28881–28883: AAC, whereas from Lombardy, those were 28881–28883: GGG.

The study then expanded to look at more than 3000 whole genome sequences from various regions around the globe and found a relationship between the presence of AAC strain in a region and the number of the COVID-19 cases there.

Data of COVID-19 cases and deaths were collected from the Statista website, Bing COVID-19 tracker, and, whenever necessary, from local government websites.

All reference sequences, including the SARS-CoV-2 reference genome NC_045512.2 (Wuhan-Hu-1), were used from the NCBI virus database.

### 2.2. Analysis

**Figure 1:**
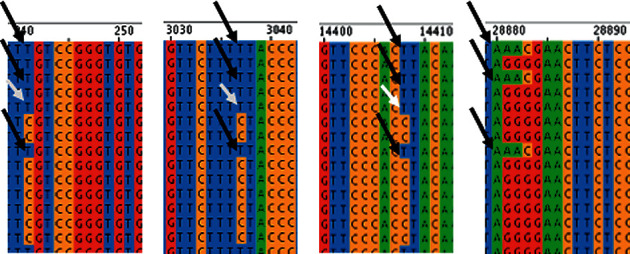
GGG > AAC change in SARS-CoV-2 is always accompanied by three other *C* > *T* mutations in positions 241, 3037, and 14408 of the virus genome as indicated by black arrows. However, *C* > *T* change in these positions does not always mean the presence in GGG > AAC as indicated by white arrows. All positions are aligned to the NCBI SARS-CoV-2 reference sequence.

The sequences downloaded from GISAID were analyzed using Jalview [8]. Jalview allowed seeing the changes in a nucleotide in a particular genomic location. As the sequences deposited by different research groups had differences in length and quality, all ∼3000 whole genome sequences were first aligned by using Clustal Omega [9]. These aligned sequences were then fed to Jalview to find every nucleotide position as shown in Figure 1.

Clusters based on various sequence features, such as on a particular nucleotide position, were built using the neighbor-joining algorithm from Jalview. This allowed clustering of the genome sequences deposited in GISAID from various geographical regions focusing on the trinucleotide sequence feature of 28881–28883: GGG and 28881–28883: AAC.

To predict the impact of a particular mutation on the protein structure, JPred secondary structure prediction service was used. PyMOL software was used to view the amino acids at a specific position in the 3D structure of the protein.

## 3. Results

### 3.1. 28881–28883: GGG > AAC Change Is a Unique Event Resulting in Two Substrains of SARS-CoV-2 Described Here as SARS-CoV-2g and SARS-CoV-2a

In all ∼3000 complete genomes of SARS-CoV-2 analyzed in this study, a bloc of trinucleotide has changed as GGG > AAC in the 28881–28883 location of the genome. All other changes in the genomes are mostly single nucleotide polymorphisms (SNPs). This observation suggests that the GGG > AAC change might have occurred at the same time or in a short span of time. Such changes would be expected to have significant impacts on the virus life cycle and pathogenicity as discussed later.

28881–28883: GGG > AAC mutation is accompanied by three other mutations, such as 241:*C* > *T*, 3037: *C* > *T*, and 14408: *C* > *T* (Figure 1), but the opposite is not always true. This implies that the 241:*C* > *T*, 3037:*C* > *T*, and 14408:*C* > *T* mutations precede the 28881–28883: GGG > AAC mutation. Among them, 14408:*C* > *T* brings 323:*P* > *L* changes in the RNA polymerase [10] of SARS-CoV-2, which may contribute to the 28881–28883: GGG > AAC change in the virus genome. Further investigation is necessary to understand this course of events.

### 3.2. SARS-CoV-2a Is a Relatively New Strain and Has a Distinct Mutation Profile Compared to SARS-CoV-2g

The 28881–28883: AAC genotype and resulting SARS-CoV-2a strain are found in samples collected in the later phase of the COVID-19 pandemic, mostly from March 2020 onward. All the sequences from Wuhan, the first epicenter of COVID-19, have the 28881–28883: GGG genotype, and so does the reference genome of SARS-CoV-2. Although one SARS-CoV-2a affected person was reported in Italy in January [11], an analysis of the sequences deposited in the GISAID database using the Nextstrain web tool (https://nextstrain.org/ncov/gisaid/global/6m) which allows to select a time window and look at the nucleotide/amino acid changes in the deposited sequences shows that the 28881–28883: GGG position has higher entropy (a measure of changes) if the sequences from December 2019 to February 2020 are compared with those from March 2020 to June 2020.

Accumulation of mutations in the 28881–28883 positions is higher after March 1, 2020, (entropy value 0.579) compared to the sequences deposited until February 2020 (entropy value 0.325).

The early SARS-CoV-2a genomes looked relatively pristine compared to SARS-CoV-2g, mostly because of the mutually exclusive mutations in them. It appears that some SARS-CoV-2 have completed a cycle of mutations to arrive at the level of SARS-CoV-2a by changing some base positions in their genome while leaving other positions untouched. SARS-CoV-2a has its own version of the leader sequence, RNA pol and nsp3, because of the complete transition in 241: *C* > *T*, 3037: *C* > *T*, and 14408: *C* > *T,* respectively. This assumption could be supported by some contemporary analyses conducted in the early months of the pandemic which showed that the mutation characteristic of SARS-CoV-2 is highly skewed towards the *C* > *U* (T) substitutions [12, 13]. Such mutation bias might have shaped the SARS-CoV-2a genotype where the 241: *C* > *T*, 3037: *C* > *T*, and 14408: *C* > *T* substitutions preceded the appearance of the 28881–28883: GGG > AAC trinucleotide changes.

**Figure 2:**
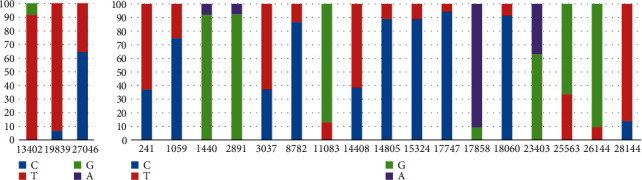
Frequency of change in base positions in SARS-CoV-2a and SARS-CoV-2g. (a) Only three positions showed more than 5% change in SARS-CoV-2a. (b) 17 positions have experienced changes in SARS-CoV-2g. *X*-axis shows base positions on reference genome, and *Y*-axis shows % occurrence of A, T, C, and G nucleotides at specific positions.

Compared to the SARS-CoV-2g counterpart, SARS-CoV-2a has very few changes in its genome. An analysis conducted on 214 SARS-CoV-2a and 1013 SARS-CoV-2g sequences from different countries shows their vivid difference. In this analysis, it was checked whether at any position of the genome, there is more than 5% change among the sequences. The result summarized in Figure 2 shows that SARS-CoV-2a has only 3 positions with such changes, whereas SARS-CoV-2g has 17 such positions. The positions which have changed in less than 10% cases generally are country-specific, except for the 26144: *G* > *T* which has been found in sequences from various countries.

Among these mutations, particularly interesting are 25563: *G* > *T* and 26144: *G* > *T* mutations that affect ORF3a and are mutually exclusive in SARS-CoV-2a and SARS-CoV-2g. This was considered important as ORF3a protein modulates the immune responses, including “cytokine storm” in the host [14]. All SARS-CoV-2a are free of those mutations, whereas in SARS-CoV-2g strain, these mutations are frequent. Interestingly, these two mutations are also mutually exclusive, i.e., all SARS-CoV-2g with 25563: *G* > *T* mutations are free from 26144: *G* > *T* mutations and vice versa.

This pattern of mutational exclusiveness requires more elaborate analysis to trace the evolution of SARS-CoV-2 strains, as they hold important clues on their pathogenicity.

### 3.3. Impacts of 28881–28883: GGG > AAC Mutation on the Pathogenicity of SARS-CoV-2

**Figure 3:**
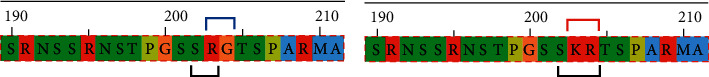
Impacts of 203–204:RG > KR mutation in the N protein. (a) The wild-type N protein with intact S-R dipeptide and (b) the mutated N protein which has the S-R dipeptide disrupted with the insertion of lysine in between them. Blue and red bars on the top indicate the wild type and mutated amino acids, respectively. Bottom black bar in (b) with asterisk indicates the disrupted S-R dipeptide.

According to the NCBI reference genome, 28881–28883: GGG > AAC bloc mutation results in two amino acids 203–204: RG > KR changes in the nucleocapsid (N) protein of SARS-CoV-2. Looking at the surrounding sequence of these amino acids (Figure 3), it appears that the mutation will discontinue a serine-arginine (S-R) dipeptide by introducing a lysine in between them.

According to the NCBI Reference Sequence: YP_009724397.2 of the SARS-CoV-2 nucleocapsid (N) protein, the changes in the mutated N protein are expected to have impacts on its structure and function. Lysine is a basic and polar hydrophilic charged (+) amino acid. Its inclusion in the motif should have an impact on the overall characteristics of the protein as reported before [15]. Especially, the serine-arginine dipeptide disruption may impact the phosphorylation of the SR-rich domain-crucial for the cellular localization and translation inhibitory function of the N protein [16].

**Figure 4:**
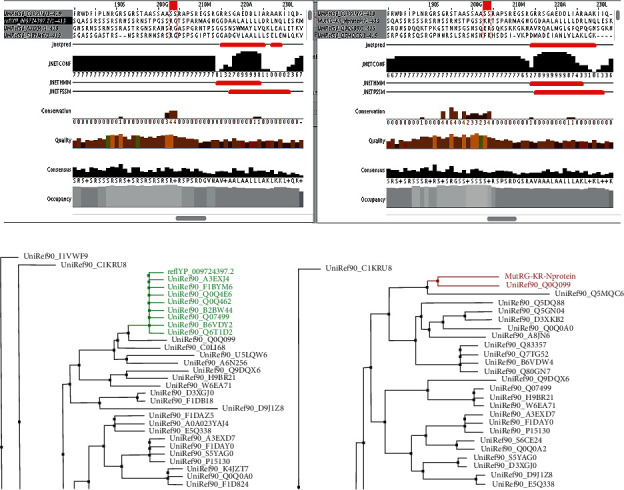
Impact of the RG > KR mutation on N protein structure. (a) The amino acid mutation at 203–204 position of the N protein changes the size and arrangement of the alpha-helix of the protein as indicated by the red bars. The left panel in the image is the reference N protein, whereas the right panel represents the mutated N protein. Conservations, quality, and consensus have been indicated at the bottom layers of the image. (b) Neighbor-joining clustering based on RG dipeptide after multiple sequence alignments shows seven proteins (green) as the neighbor of the wild type N protein. (c) Clustering based on the mutated KR dipeptide has found only one immediate neighbor (red) to the mutated N protein indicating its rarity. In both cases, the distances of the other branches indicate the different types of dipeptides found in other viruses.

Previous experimental work by deleting part of the SR domain in SARS-CoV-1 has shown reduced pathogenicity in the virus [5]. So, the disruption discussed above might have a negative impact on the mutated N protein in SARS-CoV-2. A computational analysis shows that RG > KR mutation would change the length and arrangements of the alpha-helix of the nucleocapsid protein (Figure 4). Laboratory experiments can confirm these predictions.

A multiple sequence alignment analysis and clustering based on the neighbor-joining algorithm show that changing the amino acids at 203–205: RG > KR of the N protein put SARS-CoV-2 as the only neighbor to a bat alpha-corona virus, whereas the wild type N protein clusters with several other viruses including MERS-CoV (Figure 4).

## 4. Discussion

Hundreds of mutations have been reported in SARS-CoV-2, and the tally is increasing as more sequences are deposited in the public databases. It is often a challenge to make practical use of those sequences and mutation data. This paper reports the rise and probable impacts of the strains SARS-CoV-2a and SARS-CoV-2g after analyzing available sequences and COVID-19 case data up to April 2020. The mutually exclusive nature of these two strains may work as anchors to follow them both retrospectively and prospectively.

The uniqueness of the trinucleotide mutations (28881–2883: GGG > AAC) makes it a highly potential candidate to follow the trend of the COVID-19 pandemic across regions caused by SARS-CoV-2. The molecular analysis presented in this paper has set the ground to assume that SARS-CoV-2a might be linked with the changing trends of COVID-19 cases because of the mutated SR-motif.

The SR motif of the coronaviruses is important for their pathogenic impacts on the host cells [5, 17]. By modulating the phosphorylation of the SR motif, the pathogenic ability of the virus changes. The multimerization of the N protein is crucial for its function which is modulated by phosphorylation of the SR-motif [17]. The trinucleotide bloc mutation adds a lysine instead of glycine (-SRG-to -SKR-) in the 202–204 position of the motif. Together with arginine and serine, this lysine in the motif can also be a target of phosphorylation in the infected cells [18]. It has also been shown that polyphosphorylation in PASK (polyacidic serine and lysine)-rich cluster negatively changes the function of certain enzymes [19]. This information suggests that the bloc mutation might have profound impacts on the pathogenicity of SARS-CoV-2.

Nevertheless, based on the information on the two strains of SARS-CoV-2, the severity of COVID-19 can be discussed from an immunological perspective too. Among the mutations differences between the two strains as discussed above, it is particularly important to note that the ORF3a gene in the SARS-CoV-2a strain remains unmutated compared to SARS-CoV-2g where in many cases either 25563:*G* > *A* or 26144:*G* > *A* mutations are present in a mutually exclusive manner. It is already known that ORF3a plays a critical role in inducing overreaction from inflammatory cytokines which often leads to the “cytokine storms” [20], one of the most important reasons behind the fatality from COVID-19. The complete absence of 25563: *G* > *T* and 26144: *C* > *T* mutations in SARS-CoV-2a indicates that this strain will express an active ORF3a protein, whereas more than 40% SARS-CoV-2g strains might be mutated for this gene (∼33% 25563: *G* > *T* and ∼9% 26144: *G* > *T*) (Figure 2). This extrapolation should be considered with caution as there might be other attenuating mutations and confounding factors.

However, if 28881–28883: GGG > AAC is a pivotal change that impacts the pathogenicity of SARS-CoV-2a compared to SARS-CoV-2g, then 203–204: RG > KR positions of the N protein should be targeted to design drugs to affect the replication of the virus and thus reduce the pathogenicity of SARS-CoV-2 infection. This mutation should also be considered during vaccine development. An immunoinformatic analysis has identified a strong immunodominant B cell epitope SRGGSQASSRSSSRSRNSSRNSTPGSSRGTS between 176 and 206 amino acids in the N protein sequence [19, 21]. This study has suggested that with appropriate T cell assistance, this epitope may be a good target for neutralizing antibodies and long-lived immune responses [19].

This work further recommends more active efforts to investigate the genomes of SARS-CoV-2 with closer pan-national collaboration to understand the transitions and distributions of SARS-CoV-2a and SARS-CoV-2g strains for better understanding and management of COVID-19. These interpretations based on the 28881–2883: GGG > AAC mutation need to be considered with concomitant mutations in the spike (S) proteins as they will also have profound impacts on the pathogenicity of SARS-CoV-2. The true impact of the 28881–2883: GGG > AAC trinucleotide bloc mutation might be confirmed by (i) further laboratory experiments on the particular location on the SR motif and (ii) epidemiological research by matching the sequence data from different countries with their COVID-19 patients. Factors that may contribute to the GGG > AAC change in the virus genome should also be investigated.

## Figures and Tables

**Table 1:** Size and span of the ORFs in SARS-CoV-2 according to the NCBI reference genome sequence.

ORF name	Span on the genome	Size (nt)
ORF1ab	(226–21555)	21290
S	(21563–25384)	3822
ORF3a/b	25,393–26,220	828
E	26,245–26,472	228
M	26,523–27,191	669
ORF6	27,202–27,387	116
ORF7a	27,394–27,759	366
ORF7b	27,756–27,887	132
ORF8	27,894–28,259	366
N	28,274–29,533	1260
ORF10	29,558–29,674	117

## Data Availability

All data are included within the paper.
